# Machine learning diagnostic framework for liver fibrosis in chronic hepatitis B based on routine laboratory tests

**DOI:** 10.1097/MD.0000000000047412

**Published:** 2026-03-13

**Authors:** Li Hou, Jing Tang, Wenli He, Wenpei Qin

**Affiliations:** a Medical Laboratory Center, The First Affiliated Hospital of Xinjiang Medical University, Urumqi, China.

**Keywords:** chronic hepatitis B, diagnostic model, liver fibrosis, machine learning, random forest

## Abstract

Early detection of liver fibrosis in chronic hepatitis B (CHB) patients is crucial for improving their prognosis. This study aims to develop a machine learning (ML)-based diagnostic model for evaluating the degree of liver fibrosis in patients with CHB. The present study comprised 268 patients with CHB who underwent liver biopsy from January 2022 to May 2023. Liver fibrosis was staged according to the Scheuer scoring system. The dataset is divided into a training set and a validation set in a ratio of 7.5:2.5. The feature selection process was executed through the utilization of least absolute shrinkage and selection operator regression, and 8 ML algorithms (including random forest [RF], extreme gradient enhancement, support vector machine, etc) were employed for the prediction of the performance of the significant liver fibrosis assessment model. The RF model performs best among ML models, with an area under the curve value of 0.810 for the training set and 0.793 for the validation set. Decision curve analysis indicates that the RF model exhibits the highest net benefit under most threshold probabilities. When the probability threshold is 30%, the sensitivity and net benefit are the highest, significantly outperforming other traditional scores. RF ML models can effectively assess liver fibrosis in patients with CHB. Compared with traditional indicators, they can become a safe, effective and more personalized screening method, enabling early and dynamic risk decision-making.

## 1. Introduction

Hepatitis B virus infection is a major causative agent of liver fibrosis, cirrhosis and hepatocellular carcinoma on a global scale.^[1,2]^ Hepatic fibrosis represents a pivotal phase in the progression of chronic hepatitis B (CHB) to culminate in end-stage liver disease. The timely identification and intervention of this condition are of crucial importance in order to improve the prognosis of patients. Antiviral treatment for patients with CHB has been demonstrated to not only control liver inflammation and reverse liver fibrosis, but also reduce the mortality rate related to liver cirrhosis and liver cancer.^[3-6]^

Machine learning (ML), which integrates mathematics, statistics, and computer science, enhances disease diagnosis and prognosis by intelligently learning from data to reveal hidden connections.^[7]^ The efficacy of this method in supporting clinical decision-making, predicting diseases, improving patient care and enhancing medical services has been well documented.

Liver biopsy, the gold standard for evaluating the staging of liver fibrosis, is limited in its wide application in clinical practice due to its invasive nature. Consequently, noninvasive detection methods,^[8,9]^ such as AST to platelet ratio index (APRI) and fibrosis-4 (FIB4) scoring, have gained widespread acceptance due to their ease of use and reliability. The objective of this study is to develop a novel diagnostic model by routine hematology laboratory tests and integrating modern ML techniques. The objective of this model is to accurately assess the degree of liver fibrosis in patients with CHB, thereby providing a noninvasive and efficient assessment tool for clinical practice.

## 2. Materials and methods

### 2.1. Materials

This research is in line with the Helsinki Declaration. This study was a single-center retrospective study. With the approval of the Institutional Ethics Committee of the First Affiliated Hospital of Xinjiang Medical University, the clinical data selected in this study would not affect the prognosis and privacy of patients. Therefore, the requirement for informed consent forms for patients was waived.

From January 2022 to May 2023, we retrospectively reviewed 268 patients diagnosed with CHB in our hospital. Inclusion criteria: comply with the diagnostic criteria outlined in the clinical practice guidelines for the management of hepatitis B4 in the Asia-Pacific region; not received conventional antiviral treatment; and those who have undergone liver biopsy. Exclusion criteria: coinfection with human immunodeficiency virus; combined with active hepatitis A, C, D, and E viral infection; there is a history of other conditions that lead to chronic liver disease or evidence such as alcoholic liver disease, autoimmune liver disease and drugs sexually transmitted hepatitis, etc; hepatocellular carcinoma, decompensated liver cirrhosis or combined with severe complications; accompanied by other malignant tumors and major systemic diseases; and incomplete clinical data.

### 2.2. Liver biopsy

**Figure 1.**
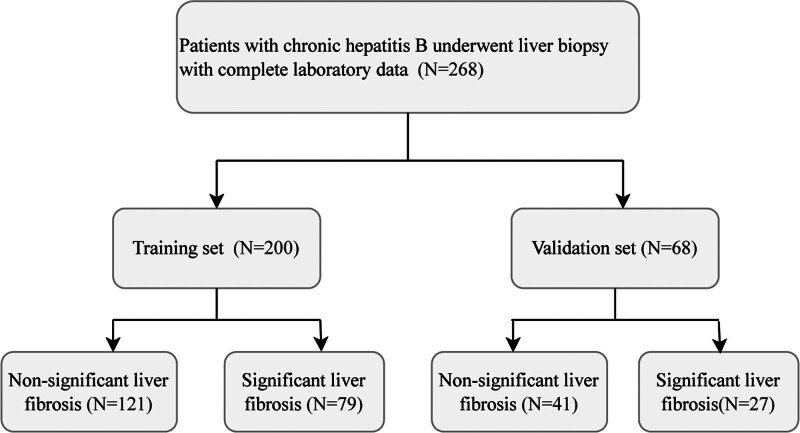
The flow chart of data enrollment and diagnosis.

Liver puncture was performed under ultrasound guidance, and the obtained liver tissue with a length of ≥15 mm was fixed in 10% formalin solution and made into conventional paraffin sections. Hematoxylin-eosin and Masson’s trichrome staining were performed. Liver fibrosis was staged according to the Scheuer scoring system: S0 (no fibrosis), S1 (portal expansion), S2 (portal fibrosis with limited septa), S3 (numerous septa with structural disorder of the lobule), and S4 (cirrhosis).^[10]^ The degree of fibrosis S0–S1 was defined as nonsignificant liver fibrosis, while S2–S4 as significant liver fibrosis. The flowchart of patient enrollment is shown in Figure 1.

### 2.3. Data collection

General patient information extracted from the electronic medical record system, as well as examination results from up to 3 days before the liver biopsy, including blood count, coagulation function tests, biochemical tests and viral serology tests. These variables as they are a routine clinical detection index, they are relatively easy to obtain and are collected for subsequent analysis purposes. They are also closely related to the severity of hepatitis B. The calculation formula for the noninvasive diagnostic model is as follows^[8,9,11]^:


$$\begin{array}{l} APRI\;=\;AST(U/L)/ASTULN(U/L)/PLT(\times 10^{9}/L)\;\times \;100 \end{array}$$

$$\begin{array}{l} FIB\text{-}4\;=\;Age\;\times \;AST(U/L)/{\left[PLT(\times 10^{9}/L)\;\times \;ALT(U/L)\right]}^{1/2} \end{array}$$

$$\begin{array}{l} GPR\;=\;GGT(U/L)/GGTULN/PLT(\times 10^{9}/L)\;\times \;100 \end{array}$$

### 2.4. Statistical analysis

The dataset is divided into a training set and a test set in a ratio of 7.5:2.5. Data that conforms to or approximately conforms to a normal distribution is described by the mean and standard deviation, and an independent sample *t*-test is used for difference analysis. In instances where data follows a non-normal distribution, the median (along with the upper and lower quartiles of each quartile) is utilized for descriptive purposes, while nonparametric tests are employed for the analysis of differences. In the context of data analysis, these values are expressed as percentages and subsequently subjected to chi-square testing.

The least absolute shrinkage and selection operator (LASSO) method, which is suitable for the reduction of high-dimensional data,^[12]^ was used to select the optimal predictive features in risk factors from the patients. Features with nonzero coefficients in the LASSO regression model were selected. Subsequently, by combining the features selected in the LASSO regression model, 8 ML algorithms were chosen to predict significant liver fibrosis and analyze the importance of the factors. The training of 8 supervised ML models was conducted and subsequently positioned within the training queue for the purpose of diagnosing advanced fibrosis. The following algorithms were included: random forest (RF), light gradient boosting machine, elastic net logistic regression, decision tree classifier, logistic regression, K-nearest neighbor, extreme gradient boosting (XGBoost), and support vector machine (SVM). A comparison was conducted of the receiver operating characteristic (ROC) curves for the RF model with those of other traditional indices, in order to evaluate its discriminative ability, a decision curve analysis (DCA) was performed in order to assess the clinical utility of the RF model. In addition, a variable importance analysis was conducted within the RF model using both a swarm plot and a bar chart to understand the contribution of each predictor to the model’s predictions. This will guide potential model refinement.

All analyses were performed using R statistical software version 4.4.2 (R Foundation, Vienna, Austria), and a 2-sided *P* < .05 was considered statistically significant.

## 3. Results

### 3.1. General characteristics

A total of 268 patients with CHB, comprising 187 males and 81 females, aged 18 to 63 years, were included in this study. The data were randomly divided into a training set and a validation set at a ratio of 7.5:2.5. There were 200 patients in the training group and 68 patients in the validation set. The difference analysis results revealed that the *P*-value of the difference analysis was >.05, as shown in Table 1.

**Table 1:** Baseline characteristics of all patients.

Variables	Total (n = 268)	Training set (n = 200)	Validation set (n = 68)	*P*	Nonsignificant liver fibrosis (n = 162)	Significant liver fibrosis (n = 106)	*P*
Gender (n, %)‡				.866			.593
Male	187 (69.78)	139 (69.50)	48 (70.59)		115 (70.99)	72 (67.92)	
Female	81 (30.22)	61 (30.50)	20 (29.41)		47 (29.01)	34 (32.08)	
Age (yr)†	38.35 ± 8.88	38.62 ± 8.55	37.53 ± 9.78	.380	37.23 ± 8.13	40.05 ± 9.70	.011
ALB (g/L)†	41.21 ± 4.46	41.27 ± 4.17	41.02 ± 5.24	.679	42.18 ± 3.98	39.72 ± 4.76	<.001
PLT (×10^9^/L)†	175.56 ± 52.29	177.55 ± 54.42	169.72 ± 45.34	.287	186.87 ± 47.81	158.29 ± 54.32	<.001
HbsAg (Iµ/mL)*	250.00 (250.00, 250.00)	250.00 (250.00, 250.00)	250.00 (250.00, 250.00)	.592	250.00 (250.00, 250.00)	250.00 (250.00, 250.00)	.324
HBV DNA (Log Iµ/mL)*	4.36 (0.48, 7.31)	4.45 (2.14, 7.43)	4.26 (0.48, 6.53)	.430	3.95 (0.48, 7.54)	5.02 (2.70, 6.91)	.367
AFU (µ/L)*	24.65 (16.39, 33.52)	24.44 (16.15, 33.52)	26.25 (17.87, 33.73)	.428	22.80 (15.35, 32.85)	27.35 (20.20, 34.15)	.013
ALP (µ/L)*	72.45 (58.75, 89.25)	72.00 (58.75, 87.00)	73.50 (60.07, 91.25)	.601	69.00 (55.25, 87.00)	76.00 (63.02, 93.00)	.018
ALT (µ/L)*	35.60 (23.15, 58.95)	35.60 (22.87, 58.50)	35.60 (24.58, 59.32)	.781	34.90 (21.40, 54.57)	37.85 (26.95, 69.77)	.019
AST (µ/L)*	26.10 (21.00, 35.73)	26.10 (20.98, 35.25)	26.10 (21.00, 37.85)	.678	23.85 (19.73, 32.15)	31.30 (23.35, 41.98)	<.001
GGT (µ/L)*	27.00 (16.00, 43.25)	26.90 (16.75, 42.00)	28.32 (16.00, 48.50)	.499	23.00 (15.00, 41.00)	31.35 (19.25, 47.50)	.006
Tbil (µmol/L)*	13.35 (10.00, 17.53)	13.60 (9.97, 17.42)	12.99 (10.17, 17.96)	.843	12.95 (9.80, 16.77)	13.92 (10.53, 18.50)	.193
TBA (µmol/L)*	4.84 (3.01, 10.03)	4.59 (2.88, 9.32)	5.71 (3.59, 12.00)	.098	4.42 (2.85, 8.06)	6.66 (3.31, 13.44)	.003
APTT*	32.60 (30.10, 34.90)	32.60 (30.00, 34.82)	32.85 (30.30, 35.05)	.554	32.15 (30.00, 34.20)	33.50 (30.73, 35.60)	.012
FIB-C*	2.82 (2.51, 3.21)	2.89 (2.52, 3.25)	2.70 (2.47, 3.05)	.145	2.92 (2.55, 3.27)	2.73 (2.40, 3.09)	.012
PT*	10.30 (9.80, 10.80)	10.30 (9.80, 10.80)	10.30 (9.87, 10.83)	.611	10.10 (9.70, 10.80)	10.40 (10.03, 11.00)	.004
GLB (µ/L)*	26.45 (23.50, 29.50)	26.40 (23.50, 29.42)	27.25 (23.48, 30.31)	.642	25.70 (22.95, 29.18)	27.70 (24.00, 30.60)	.006
HbeAg (n, %)‡				.983			.462
Negative	154 (57.46)	115 (57.50)	39 (57.35)		96 (59.26)	58 (54.72)	
Positive	114 (42.54)	85 (42.50)	29 (42.65)		66 (40.74)	48 (45.28)	

AFU = alpha-L-fucosidase, ALB = albumin, ALP = alkaline phosphatase, ALT = alanine aminotransferase, APTT = activated partial thromboplastin time, AST = aspartate aminotransferase, DNA = deoxyribonucleic acid, FIB-C = fibrinogen C domain, GGT = gamma-glutamyl transferase, GLB = globulin, HBV = hepatitis B virus, PLT = platelet count, PT = prothrombin time, TBA = total bile acid.

* Median (Q1_25%_–Q3_75%_).

† Mean ± SD.

‡ Outside parentheses is the number of cases, inside parentheses is the composition ratio (%).

### 3.2. Feature selection

**Figure 2.**
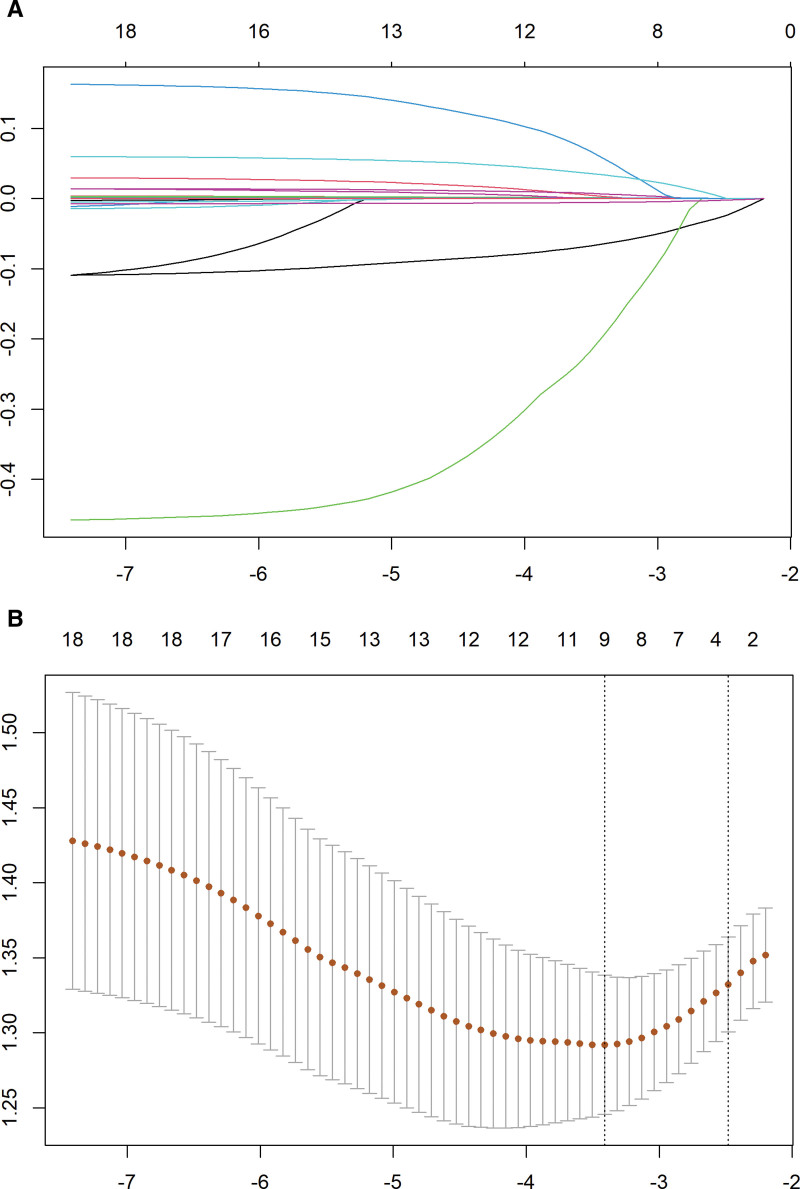
Predictor selection with LASSO regression. (A) Optimal parameter (λ) selection in the LASSO model; (B) tuning parameter (λ) selection in the LASSO model. LASSO = least absolute shrinkage and selection operator.

LASSO regression was used to screen variables among the 18 laboratory indicators and demographics in the modeling population. As shown in Figure 2A. With the change in the penalty coefficient λ, the coefficients of the independent variables initially included in the model are gradually compressed, and the coefficients of some independent variables are finally compressed to 0, which avoids overfitting of the model. The optimal penalty coefficient λ in the LASSO regression model is identified by cross-validation of 10 times the minimum criterion.^[13]^ When the λ value continues to increase to 1 standard error, the λ value is the optimal value of the model, as shown in Figure 2B. Through LASSO regression analysis, 9 variables were obtained, namely: platelet count, globulin, albumin, aspartate aminotransferase, prothrombin time, AGE, fibrosis, alpha-L-fucosidase, and alanine aminotransferase.

### 3.3. Developing of prediction model using machine learning

**Figure 3.**
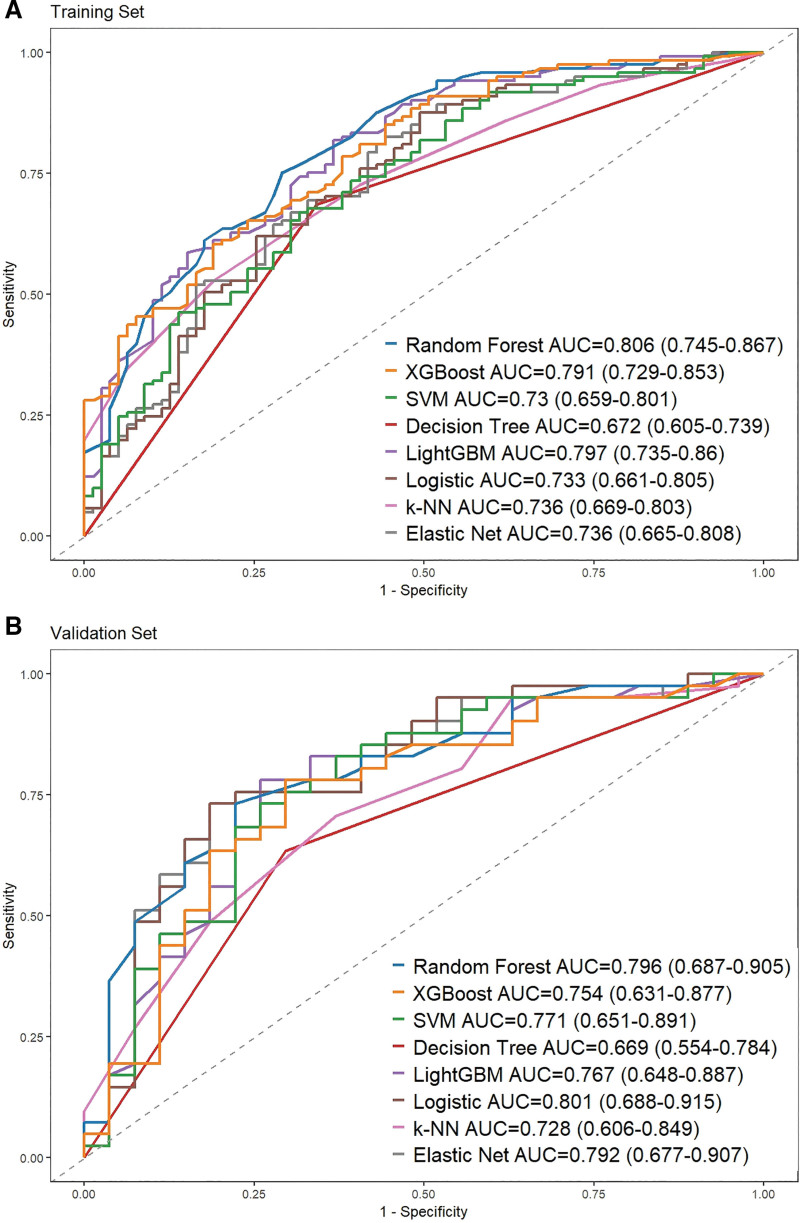
ROC curves of 8 machine learning algorithms. (A) Training set. (B) Validation set. AUC = area under the curve, ROC = receiver operating characteristic, SVM = support vector machine.

In the training cohort, 200 patients were enrolled to develop ML models. Potential predictors for model development were consistent with all variables subjected to the LASSO regression described above. Prediction algorithms used for model development included RF, light gradient boosting machine, elastic net, decision tree classifier, logistic regression, K-nearest neighbor, XGBoost and SVM. We comprehensively evaluated the performance of the models in terms of accuracy, sensitivity, specificity, positive predictive value, negative predictive value, F1 score, and area under the curve (AUC) for the 8 ML models (Table 2). The ROC curves for the 8 models in the training set (Fig. 3A) and validation set (Fig. 3B) are illustrated. In the training set and validation set, while the AUC value for RF was the highest in the validation set (AUC = 0.810) and training set (AUC = 0.793).

**Table 2:** Comparison of multiple machine learning evaluation indexes between training set and validation set.

Models	Accuracy	Sensitivity (95% CI)	Specificity (95% CI)	PPV	NPV	F1	AUC (95% CI)
Random forest*	0.765	0.868 (0.796–0.917)	0.608 (0.497–0.708)	0.772	0.750	0.817	0.810 (0.750–0.870)
Random forest†	0.735	0.805 (0.660–0.898)	0.630 (0.442–0.785)	0.767	0.680	0.786	0.793 (0.683–0.904)
XGBoost*	0.685	0.603 (0.514–0.686)	0.810 (0.710–0.881)	0.830	0.571	0.699	0.791 (0.729–0.853)
XGBoost†	0.706	0.634 (0.481–0.764)	0.815 (0.633–0.918)	0.839	0.595	0.722	0.754 (0.631–0.877)
SVM*	0.675	0.669 (0.582–0.747)	0.684 (0.575–0.776)	0.764	0.574	0.714	0.730 (0.659–0.801)
SVM†	0.721	0.707 (0.555–0.824)	0.741 (0.553–0.868)	0.806	0.625	0.753	0.771 (0.651–0.891)
Decision tree*	0.675	0.686 (0.599–0.762)	0.658 (0.548–0.753)	0.755	0.578	0.719	0.672 (0.605–0.739)
Decision tree†	0.662	0.634 (0.481–0.764)	0.704 (0.515–0.841)	0.765	0.559	0.693	0.669 (0.554–0.784)
LightGBM*	0.745	0.818 (0.740–0.877)	0.633 (0.523–0.731)	0.773	0.694	0.795	0.797 (0.735–0.860)
LightGBM†	0.750	0.780 (0.633–0.880)	0.704 (0.515–0.841)	0.800	0.679	0.790	0.767 (0.648–0.887)
Logistic*	0.730	0.876 (0.806–0.923)	0.506 (0.398–0.614)	0.731	0.727	0.797	0.733 (0.661–0.805)
Logistic†	0.706	0.805 (0.660–0.898)	0.556 (0.373–0.724)	0.733	0.652	0.767	0.801 (0.688–0.915)
KNN*	0.640	0.529 (0.440–0.616)	0.810 (0.710–0.881)	0.810	0.529	0.640	0.736 (0.669–0.803)
KNN†	0.618	0.488 (0.343–0.635)	0.815 (0.633–0.918)	0.800	0.512	0.606	0.728 (0.606–0.849)
Elastic net*	0.720	0.826 (0.749–0.884)	0.557 (0.447–0.661)	0.741	0.677	0.781	0.736 (0.665–0.808)
Elastic net†	0.691	0.756 (0.607–0.862)	0.593 (0.407–0.755)	0.738	0.615	0.747	0.792 (0.677–0.907)

AUC = area under the curve, CI = confidence interval, KNN = K-nearest neighbor, LightGBM = light gradient boosting machine, NPV = negative predictive value, PPV = positive predictive value, SVM = support vector machine, XGBoost = extreme gradient boosting.

* Training set.

† Validation set.

### 3.4. The performance of the RF model in significantly liver fibrosis

**Figure 4.**
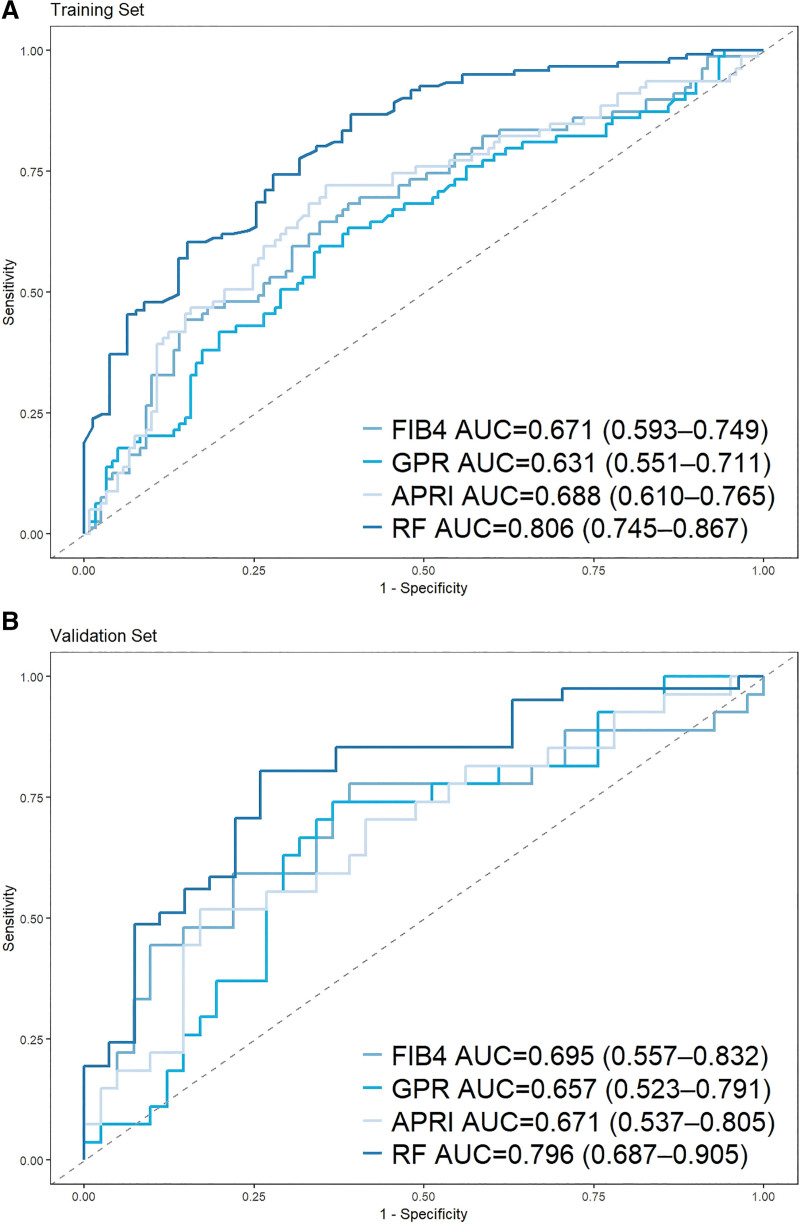
ROC comparison of the RF model with other traditional indices for significant liver fibrosis. (A) Training set. (B) Validation set. APRI = AST to platelet ratio index, AUC = area under the curve, FIB4 = fibrosis-4 score, GPR = gamma-glutamyl transpeptidase to platelet ratio, RF = random forest, ROC = receiver operating characteristic.

For diagnosing advanced fibrosis, the RF model’s AUCs were 0.810 (95% confidence interval [CI]: 0.750–0.870) in the training cohort and 0.793 (95% CI: 0.683–0.904) in the validation cohort. The AUC of the RF model is superior to other noninvasive indicators in the training set (Fig. 4A) and validation set (Fig. 4B). Superior discrimination was observed for RF versus FIB4, gamma-glutamyl transpeptidase to platelet ratio (GPR), and APRI (DeLong test, *P* = .0058, .0005, .0148), but these differences were not maintained in the validation set (*P* = .275, .126, .171).

**Figure 5.**
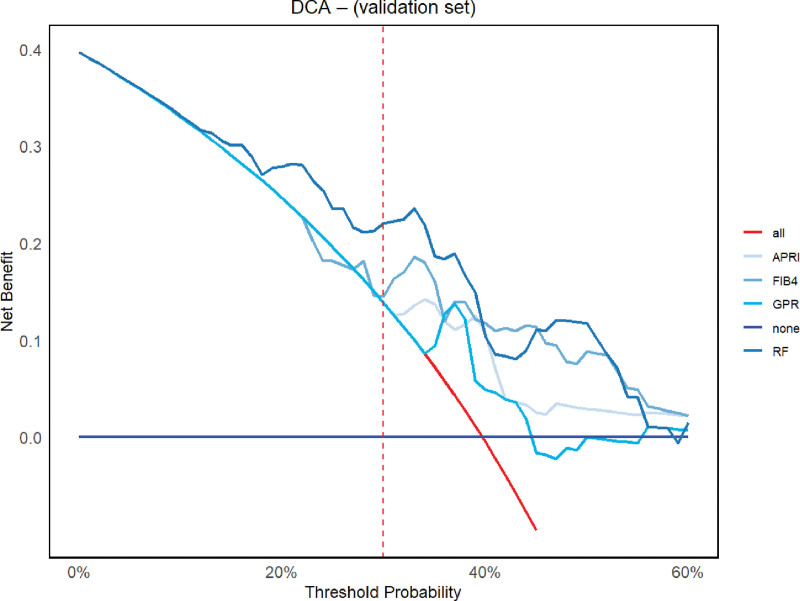
Decision curve analysis for RF model and other traditional indices in validation set. APRI = AST to platelet ratio index, DCA = decision curve analysis, FIB4 = fibrosis-4 score, GPR = gamma-glutamyl transpeptidase to platelet ratio, RF = random forest .

The horizontal axis of the DCA graph thus represents the threshold probability, whilst the vertical axis denotes the net benefit to patients of receiving treatment. As demonstrated in Figure 5, the average net benefit of the RF model was the most significant at 0.208, with a substantial margin over competing models such as APRI (0.174), FIB4 (0.192), and GPR (0.162). Within the entire threshold probability range of 0.30 to 0.60 on the horizontal axis of the decision curve, the RF model consistently generates the highest net return (Table 3). When the threshold probability is set at 30%, the RF net benefit attains 0.221, which is 53% higher than that of FIB4 at the equivalent threshold. The findings indicate that RF has the capacity to facilitate superior decision-making processes under diverse clinical risk profiles and possesses the capability to inform personalized treatment decisions.

**Table 3:** Comparison of diagnostic performance and clinical net benefits in validation set.

Probability threshold	Cutoff	Model	Sensitivity (95% CI)	Specificity (95% CI)	PPV	NPV	Youden index	Net benefit
30%	0.300	RF	1.000 (0.914–1.000)	0.037 (0.007–0.183)	0.612	1.000	0.037	0.221
	0.783	FIB4	0.366 (0.236–0.519)	0.778 (0.592–0.894)	0.714	0.447	0.144	0.145
40%	0.300	RF	1.000 (0.914–1.000)	0.037 (0.007–0.183)	0.6119	1.000	0.037	0.103
	1.373	FIB4	0.780 0.633–0.880)	0.519 (0.340–0.693)	0.711	0.609	0.299	0.118
50%	0.300	RF	0.951 (0.839–0.987)	0.370 (0.215–0.558)	0.696	0.833	0.321	0.118
	1.914	FIB4	0.927 (0.806–0.975)	0.333 (0.186–0.522)	0.679	0.750	0.260	0.088
60%	0.300	RF	0.854 (0.716–0.931)	0.407 (0.245–0.593)	0.686	0.647	0.261	0.015
	2.462	FIB4	0.927 (0.806–0.975)	0.222 (0.106–0.408)	0.644	0.667	0.149	0.022

CI = confidence interval, FIB4 = fibrosis-4 score, NPV = negative predictive value, PPV = positive predictive value, RF = random forest.

### 3.5. The importance variable of RF model

**Figure 6.**
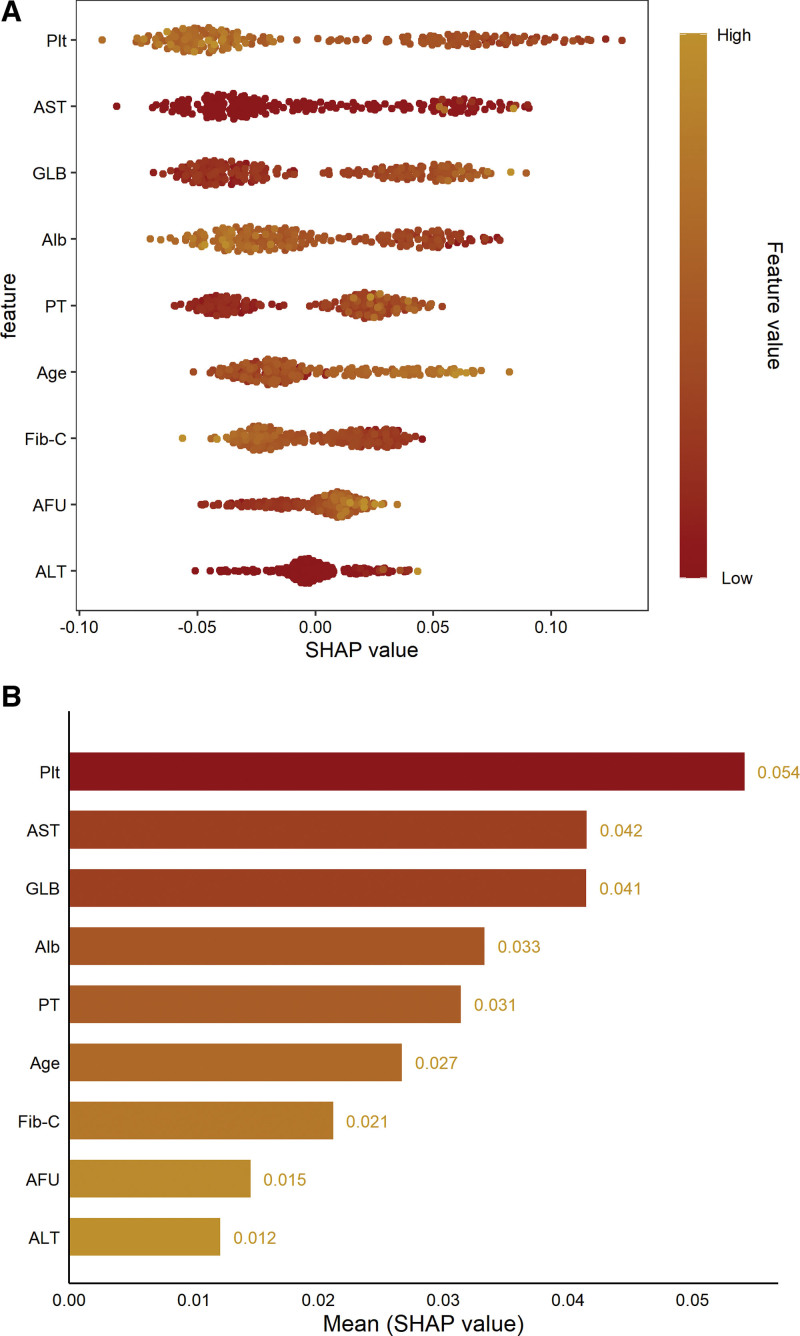
Variable importance analysis in random forest model. (A) Swarm plot; (B) bar chart. ALB = albumin, FIB = fibrosis , SHAP = shapley additive explanations.

As the AUC value of RF was higher than other ML models, indicating good predictive power. The importance of different variables were showed as which indicated the degree of contribution to the RF prediction model. All variables were ranked from the RF analysis (Fig. 6).

## 4. Discussion

Hepatitis B virus infection can trigger immune responses and liver cell damage, leading to inflammation and fibrosis, and may eventually develop into liver cirrhosis or liver cancer.^[14]^ Early identification of liver pathological changes is crucial for guiding antiviral treatment, and timely intervention can reduce the risk of adverse outcomes.^[15,16]^

This study aims to provide a noninvasive and efficient assessment method for the degree of liver fibrosis in patients with CHB by constructing a ML model based on routine laboratory testing items. In this study, we employed multiple ML algorithms, including RF, XGBoost, SVM, etc, to predict the degree of liver fibrosis in patients with CHB. By comparing the performance of these models, we found that the RF model performed the best in terms of accuracy, sensitivity, specificity, positive predictive value, negative predictive value, F1 score and AUC. AUC for the training set and validation set are 0.810 and 0.793, respectively. The efficacy of this metric has been demonstrated by its superior performance in comparison to other noninvasive metrics, including APRI, FIB4, and GPR. This finding suggests that the radio frequency model is capable of extracting key features from high-dimensional data and utilizing these features for accurate predictions. This finding is consistent with contemporary research trends, thereby further validating the practical benefits and advantages of ML methods in this domain.^[17,18]^The comparison of ROC curves further confirmed the superiority of the RF model in the diagnosis of significant liver fibrosis. In both the training set and the validation set, AUC of the RF model were higher than those of other noninvasive tests. This finding indicates that the RF model can effectively distinguish different degrees of liver fibrosis on various datasets. In the training set, the RF model dem”nstr’ted significant performance disparities in comparison to other noninvasive tests through the DeLong test (*P* < .05). However, in the validation set, these differences did not reach statistical significance (*P* > .05). This finding indicates that, despite the efficacy of RF models in the training set, their capacity for generalization in independent datasets may be constrained. The absence of statistical significance observed in the validation set may be attributable to the limited sample size, which constrained the capacity of statistical tests. Consequently, future research should be conducted on larger-scale independent datasets to verify the generalization ability and stability of RF models.

Despite the DeLong test demonstrating no statistically significant difference in AUC between the RF model in the validation set and the traditional score (*P* > .05), the DCA revealed clear clinical advantages. When compared with other traditional noninvasive indicators, at a- 30% threshold probability, the RF sensitivity reached 1.000 (95% CI: 0.914–1.000), and the net gain was 0.221. This performance indicates that the RF model rarely misidentifies positive cases and is suitable for excluding the disease in the population of CHB patients. The system’s precision ensures that the vast majority of patients are correctly identified and released. The RF model has the capacity to furnish clinicians with quantitative instruments for the early identification of disease. Clinicians may employ the RF 30% threshold for the purpose of exclusion screening, thereby rendering it a more sensitive, dynamic and individualized quantitative tool to support clinical decision-making. The importance of variables in the RF model indicates that platelet count, aspartate aminotransferase, and globulin are regarded as key factors in pre”icti’g liver fibrosis. These variables have also been identified as important predictors of liver fibrosis progression in other studies,^[19-21]^ further verifying the reliability of our model analysis.

Although this study successfully constructed and verified a ML-based diagnostic model, providing a new noninvasive method for the assessment of liver fibrosis in patients with CHB, it also has limitations. As a single-center retrospective study, the sample size was limited, which might have restricted the generalization of the model. In addition, the model mainly relies on routine detection items and may not include all relevant potential factors. Future research needs to validate the model in larger-scale multicenter cohorts and explore more biomarkers to enhance the accuracy and applicability of the model.

## 5. Conclusions

In comparison with conventional indicators, this machine model is anticipated to function as a reliable instrument for the screening of liver fibrosis in CHB, thereby providing clinical practice with a quantitative screening tool that is both safe and efficient, and supporting early, dynamic and individualized decision-making.

## Acknowledgments

We thank all the participants for their commitment to this study.

## Author contributions

**Conceptualization:** Li Hou, Jing Tang.

**Data curation:** Wenpei Qin.

**Formal analysis:** Wenpei Qin.

**Software:** Li Hou.

**Supervision:** Wenli He.

**Validation:** Wenli He.

**Writing – original draft:** Li Hou.

**Writing – review & editing:** Li Hou.
